# Comparison of the efficacy and invasiveness of manual and automated gonioscopy

**DOI:** 10.1371/journal.pone.0284098

**Published:** 2023-04-06

**Authors:** Yuki Takagi, Mitsunori Watanabe, Takashi Kojima, Yukihiro Sakai, Ryo Asano, Kazuo Ichikawa

**Affiliations:** 1 Chukyo Hospital 1–10, Sanjo 1-chome, Mina-ku, Nagoya, Aichi, Japan; 2 Chukyo Eye Clinic 12–22, Sanbonmatsu-cho, Atsuta-ku, Nagoya, Aichi, Japan; 3 Nagoya Eye Clinic 24–14, Namiyose-cho, Atsuta-ku, Nagoya, Aichi, Japan; 4 Asano Eye Clinic, 3009, Iriba 1-chome, Minato-ku, Nagoya, Aichi, Japan; Yamagata University Faculty of Medicine: Yamagata Daigaku Igakubu Daigakuin Igakukei Kenkyuka, JAPAN

## Abstract

**Purpose:**

To compare the efficacy and invasiveness of manual gonioscopy and automated 360-degree gonioscopy.

**Method:**

Manual and automated gonioscopy were performed on 70 patients with glaucoma. Manual gonioscopy was performed by a glaucoma specialist and an ophthalmology resident, and automated gonioscopy (GS-1) was performed by orthoptists. We compared the examination time for acquiring gonioscopic images (GS-1: 16 directions; manual gonioscopy: 8 directions). Furthermore, we compared the pain and discomfort scores during the examination using the Individualized Numeric Rating Scale. Among the images acquired by automated gonioscopy, we also evaluated the percentages of acquired images that could be used to determine the angle opening condition.

**Results:**

The examination time was not significantly different between manual (80.2±28.7) and automated gonioscopy (94.7±82.8) (p = 0.105). The pain score of automated gonioscopy (0.22±0.59) was significantly lower than that of manual gonioscopy (0.55±1.11) (p = 0.025). The discomfort score was not significantly different between manual (1.34±1.90) and automated gonioscopy (1.06±1.50) (p = 0.165). Automated gonioscopy successfully acquired clear gonioscopic images in 93.4% of the total images.

**Conclusion:**

Automated gonioscopy is comparable in examination time and invasiveness to manual gonioscopy and may be useful for 360-degree iridocorneal angle evaluation.

## Introduction

Gonioscopy was developed in the 1800s [1] and is essential for glaucoma examination. In the evaluation of glaucoma, it is necessary to differentiate open-angle glaucoma from closure glaucoma [2, 3]. Since angle-closure glaucoma is more common in Asians, including Japanese, than in Europeans [4], gonioscopy is highly important in Asian countries.

Manual gonioscopy is useful in both dynamic and static examination [5]. However, there are several issues, such as the learning curve of manual gonioscopy and disagreement between inter- and intra-observer agreement [5, 6]. Because gonioscopy requires direct contact with the cornea and it is difficult to acquire precise 360-degree gonioscopic images, reports suggested that gonioscopic examination is not performed often in ophthalmology clinical settings [7, 8].

As alternatives to gonioscopy, anterior segment optical coherence tomography (AS-OCT) and ultrasound biomicroscopy (UBM) can be performed noninvasively and have been found to be useful in the evaluation of iridocorneal angle structures [9–12]. In particular, AS-OCT appears to be highly useful because of its short examination time. However, AS-OCT and UBM cannot evaluate trabecular meshwork pigmentation or differentiate whether angle closure is organic or functional. It has been reported that AS-OCT tends to identify angle closures more frequently than gonioscopic examination [12–14] but may not match the findings of gonioscopic examination in a certain number of cases [13, 15]; therefore, it has yet to completely replace gonioscopic examination.

A GS-1 gonioscope camera (NIDEK Co. Gamagori, Japan) was able to acquire 360-degree gonioscopic photographs automatically. Although the examination time of GS-1 and the quality of the GS-1 images have been reported previously [16–18], there is a paucity of information on the comparison of the efficacy between manual gonioscopy and GS-1.

In this study, we compared the examination time and invasiveness between automated gonioscopy performed by orthoptists and manual gonioscopy performed by a glaucoma specialist and an ophthalmology resident. In addition, we evaluated the quality of the acquired photographs by automated gonioscopy and the learning curve of the automated gonioscopic examination.

## Materials and methods

### Patient and study design

The study group included 70 glaucoma patients who visited the glaucoma outpatient clinic of the Chukyo Eye Clinic from June 2019 to June 2020. All patients were Japanese. The patients had already undergone gonioscopy several times before participating in the study.

Manual gonioscopy was performed by a glaucoma specialist (M. W.) and an ophthalmology resident (Y. T.). Automated gonioscopy and a questionnaire were administered immediately after the examination. Automated gonioscopy (GS-1) was performed by orthoptists. The gonioscopic examination time was defined as the duration from the time the gonioscope touched the cornea to the end of the examination.

We administered a questionnaire regarding pain and discomfort during the examination and compared the examination time and subjective scores between manual gonioscopy and GS-1. In addition, we evaluated the quality of the photographs acquired using automated gonioscopy.

The institutional review board of the Chukyo Eye Clinic approved the research protocol (20190424–01). Written informed consent was obtained from all patients. This study adhered to the tenets of the Declaration of Helsinki.

### Manual gonioscopy

After applying 0.4% oxybuprocaine eye drops (Benoxil ophthalmic solution, Santen, Osaka), a gonioscope was applied to the cornea. We acquired gonioscopic photographs in eight directions (nasal, temporal, superior, inferior, nasal-superior, nasal-inferior, temporal-superior, temporal-inferior) with the attached camera of the slit lamp (THD-341F, 410000 pixels, Ikegami Tsushinki Co. Ltd., Osaka). We measured the examination time using stopwatches to acquire gonioscopic photographs in eight directions. We used a Sussman four-mirror hand-held gonioscope large ring (Ocular Instruments, Bellevue, WA) and did not use hydroxyethyl cellulose (Scopizol, Senju, Osaka).

### Automated gonioscopy (GS-1)

Automated gonioscopy (GS-1) examinations were performed by four orthoptists. After applying anesthetic eye drops, GS-1 was applied to the cornea. We measured the examination time by using stopwatches to acquire GS-1 photographs after GS-1 touched the eyes. Furthermore, we classified the quality of the acquired photographs of automated gonioscopy: Grade 0, clear and focused images that enable easy identification of the angle structure; Grade 1, images were slightly blurred but still allowed for identification of the angle structure; and Grade 2, blurred images that make it difficult to identify the angle structure.

### Evaluation of pain and discomfort scores

We compared the pain and discomfort scores during manual gonioscopy and GS-1 examinations. Using the Individualized Numeric Rating (INR) scale, each item was scored from 0 (no pain/discomfort) to 10 (very unbearable pain/discomfort).

### Comparison of manual and automated gonioscopy

Manual gonioscopy was performed by a glaucoma specialist (M. W.) with more than 15 years of experience and an ophthalmology resident (Y.T.) with 3 years of experience. Thirty-six patients were examined by M. W., and the remaining 34 patients by Y.T. GS-1 was performed by four orthoptists with varying years of experience. The examination time, pain score, and discomfort score obtained by the glaucoma specialist, ophthalmology resident, and orthoptists were compared.

### Evaluation of the learning curve of automated gonioscopy

To evaluate the learning curve of GS-1, we compared the examination time, pain and discomfort scores of GS-1 between the first and last ten cases that were examined by the same orthoptist who performed GS-1 for more than 30 cases. In addition, we compared the clinical characteristics between the two groups, for which the examination lasted for 1 min or longer (long time group) and less than 1 min (short time group).

### Statistical analysis

The Mann-Whitney test was performed to compare the parameters obtained by the glaucoma specialist, ophthalmology resident, and orthoptists. The correlation between the examination time of GS-1 and clinical characteristics was analyzed using Pearson’s test. The t-test was used to compare the parameters between the groups in which the examination time of GS-1 was 1 min or longer and less than 1 min. Statistical analyses were performed using Prism 5 (GraphPad Software, San Diego, CA) and Excel 2016 (Microsoft, Washington, DC). A p-value of less than 5% was considered statistically significant.

## Results

**Fig 1:**
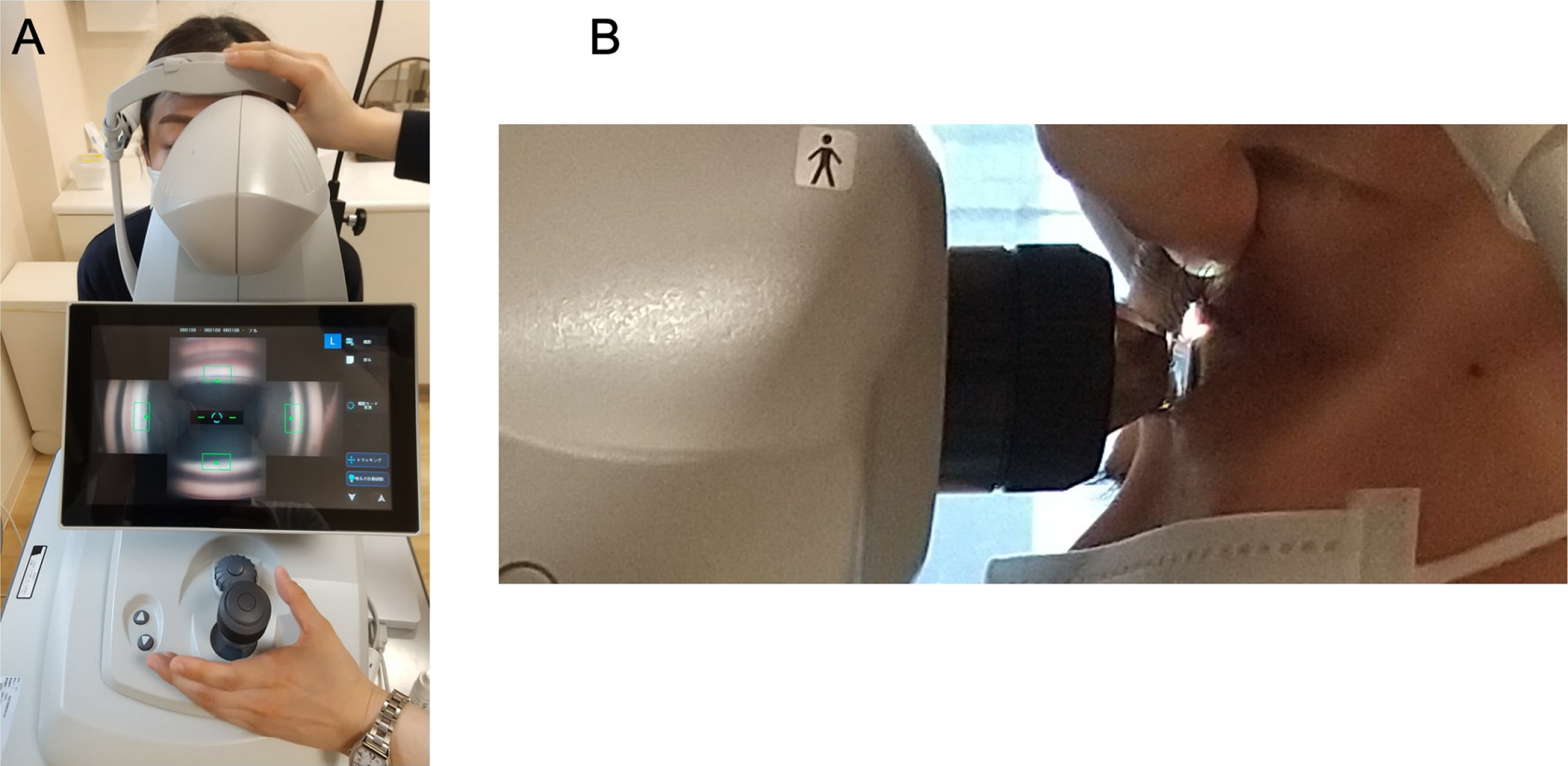
The images during a GS-1 examination. Panel A shows the monitor of GS-1 during the examination. The screen displays angle images in four directions in real-time, allowing the examiner to check whether the image is in focus. Panel B shows the GS-1 examination from the side. Lenses are large and require a wide opening of the eyelids.

**Fig 2:**
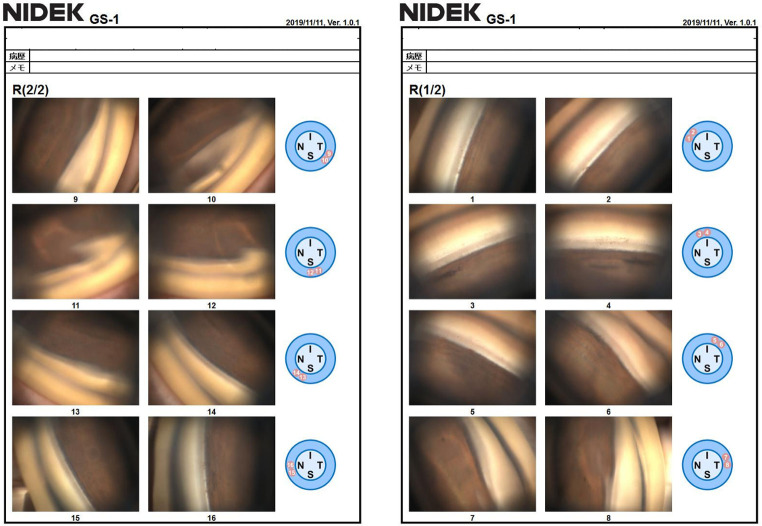
Representative GS-1 gonioscopic images in normal eyes. High-quality gonioscopic images in 16 directions are shown.

The study group included 70 glaucoma patients. Table 1 shows the patients’ demographic characteristics. The mean patient age was 66.8±12.1 years, 38 (54.3%) were women, the right eye was examined in 65.7% (46 eyes) of the cases, and 74.3% (52 eyes) of the cases were phakic. Figs 1 and 2 show images of an examination using GS-1 and its typical results, respectively.

**Table 1:** Demographics of the patients.

Characteristics	Total (n = 70)
Age, years	66.8±12.1
Sex (female, male), number (%)	Female: 38 (54.3%), male: 32 (45.7%)
Number of right eyes (%)	46 (65.7%)
Lens status, number of phakic eyes (%)	52 (74.3%)
Examined eye	
Best**-**corrected visual acuity (logMAR)	0.097±0.24
Intraocular pressure (mmHg)	16.6±5.9
Visual field, mean deviation (dB)	-11.6±7.8
Spherical equivalent (D)	-2.9±3.8
Non-examined eye	
Best**-**corrected visual acuity (logMAR)	0.067±0.29
Intraocular pressure (mmHg)	16.1±6.1
Visual field, mean deviation (dB)	-10.1±9.6
Spherical equivalent (D)	-2.7±3.7
Type of glaucoma	
POAG, number of patients (%)	41 (58.6%)
PACG, number of patients (%)	5 (7.1%)
SOAG, number of patients (%)	24 (34.3%)

logMAR: the logarithm of the minimum angle of resolution, D: diopter, POAG: primary open-angle glaucoma, PACG: primary angle-closure glaucoma, SOAG: secondary open-angle glaucoma. The values are mean ± standard deviation.

### Learning curve for automated gonioscopy evaluation

We compared the examination time of automated gonioscopy between the first and last ten cases. The examination time of the last ten cases (75.0±91.9 s) was significantly shorter than the first ten cases (101.0±85.3 s) (p = 0.048).

### Comparison of automated gonioscopy and manual gonioscopy

The results of the comparison between manual and automated gonioscopy are presented in Table 2. The examination time was not significantly different between manual (80.2±28.7 s) and automated gonioscopy (94.7±82.8 seconds) (p = 0.105). The pain score (0.22±0.59 s) of automated gonioscopy was significantly lower than that of manual gonioscopy (0.55±1.11 pts) (p = 0.025). The discomfort score was not significantly different between manual (1.34±1.90 pts) and automated gonioscopy (1.06±1.50 pts) (p = 0.165).

**Table 2:** Comparison of the examination time, pain score, and discomfort score between manual gonioscopy and automated gonioscopy (GS-1).

	Manual gonioscopy	GS-1	p-value
**Examination time (s)**	80.2±28.9 (CV: 0.36)	94.7±83.5 (CV: 0.87)	0.079
**Pain score (points)**	0.55±1.11 (CV: 2.02)	0.22±0.59 (CV: 2.70)	0.053
**Discomfort score (points)**	1.34±1.90 (CV: 1.42)	1.06±1.50 (CV: 1.42)	0.49

CV: coefficient of variation

### Comparison of examination time

**Fig 3:**
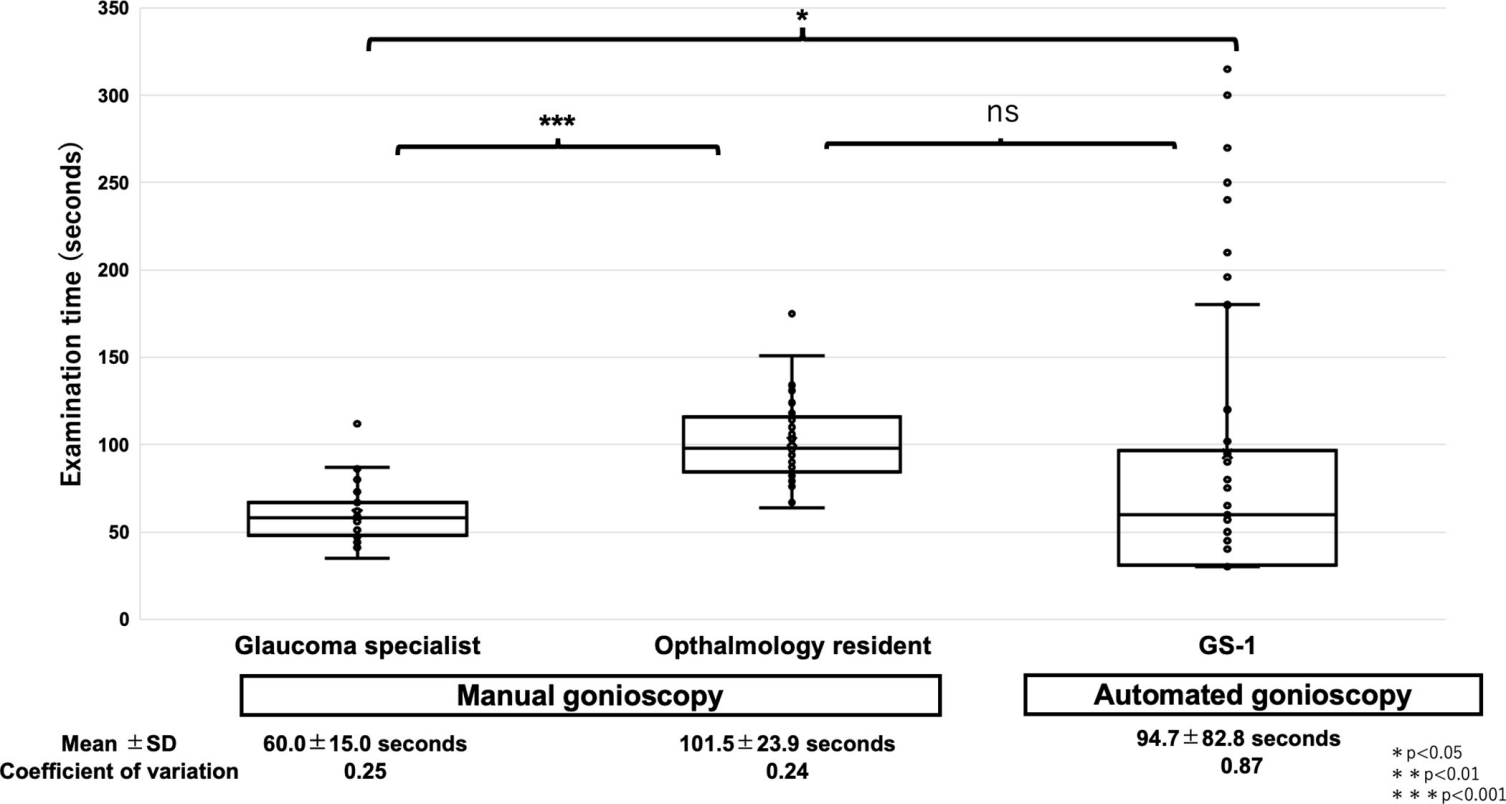
Analysis of the examination time between the glaucoma specialist, ophthalmology resident, and automated gonioscopy. *, **, and *** represent p<0.05, p<0.01, and p<0.001, respectively. ns represents statistically not significant.

We compared the examination time between manual gonioscopy by the examiner and automated gonioscopy, as shown in Fig 3. The examination time for the glaucoma specialist (60.0±15.0 s) was shorter than that for the ophthalmology resident (101.5±23.9, p<0.0001) and automated gonioscopy (94.7±82.8 s, p = 0.0168). There was no significant difference in examination time between the ophthalmology resident and automated gonioscopy (p = 0.65).

### Comparison of pain scores

**Fig 4:**
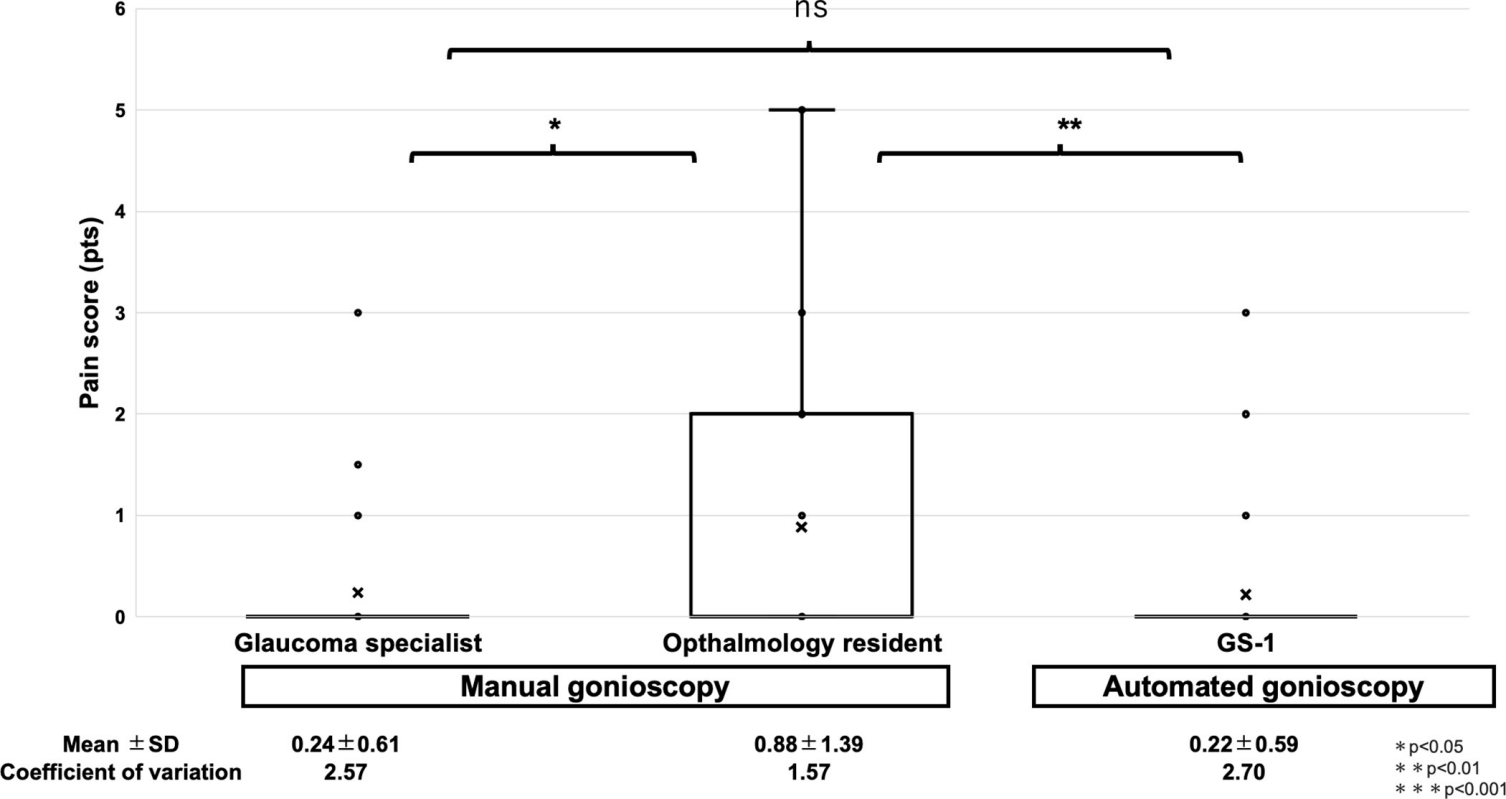
Comparison of the pain scores during examinations between the glaucoma specialist, ophthalmology resident, and automated gonioscopy. *, **, and *** represent p<0.05, <0.01, and <0.001, respectively. ns represents statistically not significant.

We compared the pain score between manual gonioscopy by the examiner and automated gonioscopy (Fig 4). The pain scores in evaluations by the glaucoma specialist, ophthalmology resident, and automated gonioscopy were 0.24±0.61, 0.88±1.39, and 0.22±0.59, respectively. The pain scores for the glaucoma specialist examinations were significantly lower than those for the ophthalmology resident examinations (p = 0.026). In addition, the pain scores for the automated gonioscopy were significantly lower than that for the ophthalmology resident examinations (p = 0.0037). There was no significant difference in pain scores between the glaucoma specialist and automated gonioscopy evaluations (p = 0.80).

### Comparison of discomfort scores

**Fig 5:**
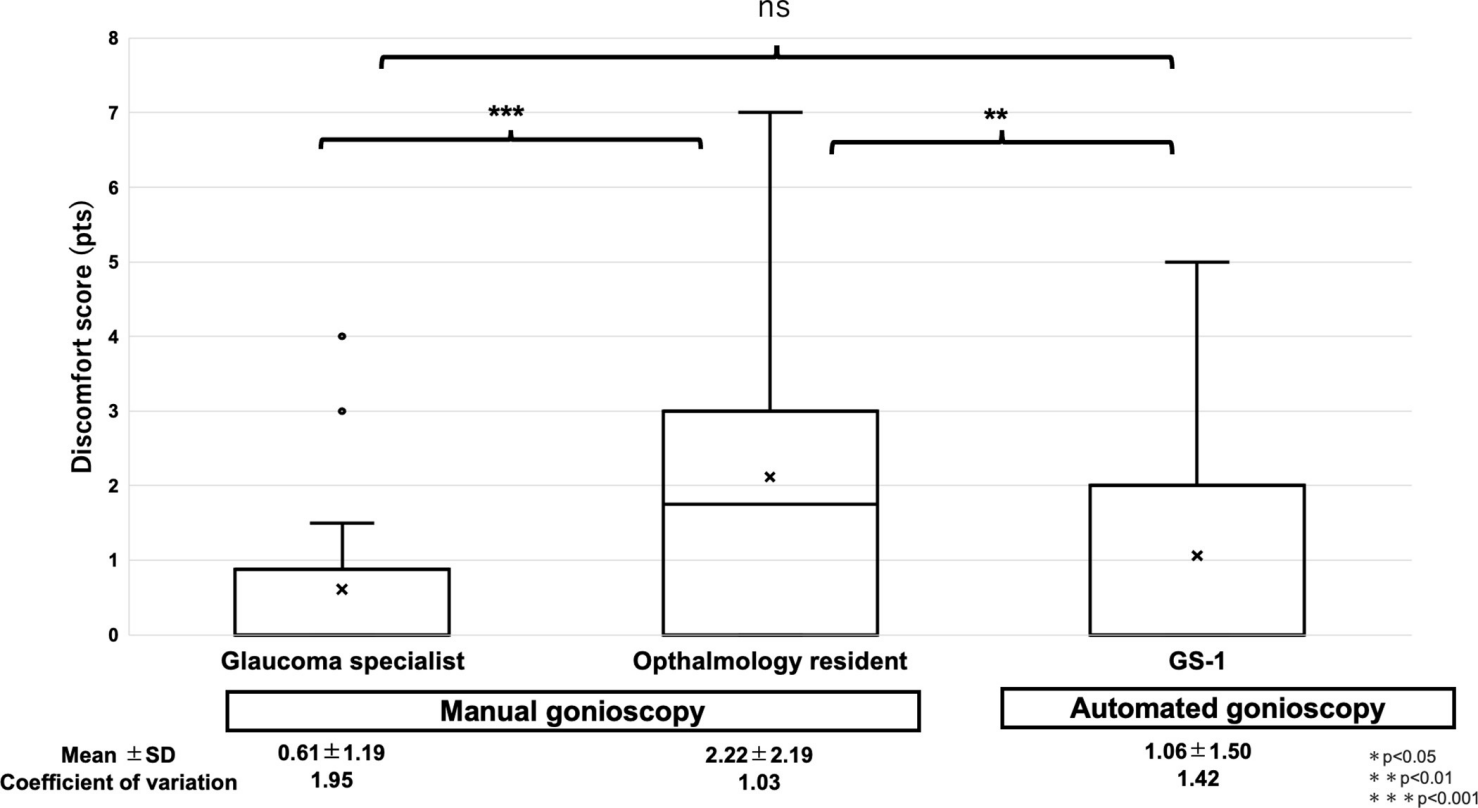
Analysis of the discomfort scores in the examinations by the glaucoma specialist, ophthalmology resident, and automated gonioscopy. *, **, and *** represent p<0.05, <0.01, and <0.001, respectively. ns represents statistically not significant.

We compared the discomfort scores between manual gonioscopy by the examiner and automated gonioscopy, as shown in Fig 5. The discomfort scores in evaluations by the glaucoma specialist, ophthalmology resident, and automated gonioscopy were 0.61±1.19, 2.22±2.19, and 1.06±1.50, respectively. The discomfort scores for the glaucoma specialist examinations were significantly lower than those for the ophthalmology resident examinations (p = 0.0003). In addition, discomfort scores in automated gonioscopy were significantly lower than those in ophthalmology resident examinations (p = 0.0078). There was no significant difference in discomfort scores between the glaucoma specialist and automated gonioscopy evaluations (p = 0.11).

### Quality assessment of automated gonioscopy images

There were a total of 1120 automated gonioscopy photographs. Among them, 986 images were defined as Grade 0 (clear and focused), 60 as Grade 1 (slightly blurred), and 74 as Grade 2 (blurred). Of all images, 93.4% were Grade 0 or 1.

### Evaluation of the factors influencing the examination time of automated gonioscopy

The correlation between the examination time of automated gonioscopy and visual acuity, visual field, and refractive error of the examined and non-examined eyes were analyzed (Table 3). Examination time showed a low positive correlation with visual acuity in the examined eye (r = 0.24, p = 0.043). In addition, we compared the clinical characteristics between the long- and short-time groups (Table 4). There were no significant differences between the two groups.

**Table 3:** Correlation between the examination time of GS-1 and clinical characteristics of the patients.

Characteristics	Correlation	p-value
Examined eye		
Best**-**corrected visual acuity (logMAR)	0.24	0.043
Intraocular pressure (mmHg)	-0.058	0.64
Visual field, mean deviation (dB)	-0.18	0.15
Spherical equivalent refractive error, diopters (D)	0.15	0.23
Non-examined eye		
Best**-**corrected visual acuity (logMAR)	0.0014	0.99
Intraocular pressure (mmHg)	-0.13	0.29
Visual field, mean deviation (dB)	-0.056	0.65
Spherical equivalent refractive error, diopters (D)	0.048	0.47

logMAR: the logarithm of the minimum angle of resolution

**Table 4:** Subgroup comparison of clinical characteristics of the patients between examinations lasted for <1 min and ≥ 1 min.

Characteristics	Examination time: < 1 min	Examination time: ≥ 1 min	p-value
Examined eye			
Best**-**corrected visual acuity (logMAR)	0.081±0.20	0.13±0.30	0.42
Intraocular pressure (mmHg)	16.5±5.2	16.5±6.9	0.97
Visual field, mean deviation (dB)	-10.9±7.9	-13.0±7.7	0.35
Spherical equivalent refractive error, diopters (D)	-3.1±4.1	-2.4±3.4	0.47
Non-examined eye			
Best**-**corrected visual acuity (logMAR)	0.033±0.20	0.13±0.4	0.20
Intraocular pressure (mmHg)	16.0±3.4	16.0±8.9	0.96
Visual field, mean deviation (dB)	-10.7±10.0	-9.1±8.9	0.57
Spherical equivalent refractive error, diopters (D)	-2.8±4.0	-2.6±3.2	0.86

logMAR: the logarithm of the minimum angle of resolution. The values are mean ± standard deviation.

## Discussion

Gonioscopy is essential for glaucoma examination. However, the use of gonioscopy is limited because of the complexity of the examination and the skills required. Gonioscopy is an invasive examination because it needs to touch the cornea directly. In addition, it is difficult to acquire precise 360-degree gonioscopic images using manual gonioscopy. GS-1 can acquire 360-degree gonioscopic images automatically and complete the examination in a short time [17]. However, to the best of our knowledge, no studies have evaluated pain and discomfort during automated gonioscopy. In this study, we compared the examination time and invasiveness between automated and manual gonioscopy. Moreover, the learning curve of automated gonioscopy was evaluated.

In our study, automated gonioscopy lasted 94.7±83.5 s. However, in a previous report [17], automated gonioscopy was reported to take approximately 20 s from GS-1 touching the eyes to the acquisition of the automated gonioscopy photographs. This discrepancy in the study results may be because our study included many cases of advanced glaucoma, which required more examination time due to poor eye fixation. In the previous study, the authors performed pre-training for approximately one week, which might have affected the examination time and may be another reason for the shorter examination time in their study. In our study, there was a significant trend toward shorter examination times in the latter half of GS-1 examinations by the same examiner, suggesting the existence of a learning curve in the test.

Although there was no significant difference in measurement time between automated and manual gonioscopy, automated gonioscopy showed a larger deviation in examination time. The deviation of examination time for manual gonioscopy was 28.9 s, with a coefficient of variation of 0.36, which was not large, and it was assumed that all cases, including those with poor eye fixation, could be examined in a similar amount of time. On the other hand, the deviation of examination time for automated gonioscopy was 83.5 s, with a larger coefficient of variation of 0.87, which suggested that automated gonioscopy had a large variation in examination time depending on the eye fixation condition. Manual gonioscopy can control the position of the eyeball because the gonioscope is in direct contact with the eye; therefore, the examination time of manual gonioscopy fluctuated little and was generally performed in approximately the same duration. However, automated gonioscopy cannot control the position of the eyeballs and relies on the examiner’s fixation effort, which is assumed to be the reason for the large fluctuation in the examination time.

The examination time, pain score, and discomfort score were significantly lower in glaucoma specialist examinations than in ophthalmology resident examinations. It was assumed that the glaucoma specialist was more proficient in manual gonioscopy, which allowed him to perform the examination in a shorter time and less invasively. This suggests that the examiner’s experience and skills affect manual gonioscopy. Regarding the comparison between the glaucoma specialist and automated gonioscopy, only the examination time was shorter in glaucoma specialist examinations. In the comparison between the ophthalmology resident and automated gonioscopy examinations, while the examination time was not significantly different, the pain and discomfort scores were significantly lower in automated gonioscopy. This suggests that automated gonioscopy can be performed in a shorter time and is less invasive than when performed by inexperienced ophthalmologists. In addition, automated gonioscopy requires more examination time than glaucoma specialists and can be performed with the same level of invasiveness. Automated gonioscopy can prevent further pressure to the eye once a certain amount of pressure is applied to the lens, which might lead to lower pain and discomfort scores than in manual gonioscopy. In this study, we used a gonioscope without flanges. Manual gonioscopy with a flanged gonioscope requires hydroxyethyl cellulose, which may cause higher levels of discomfort to the examinee. This suggests that GS-1 is less invasive than manual gonioscopy.

Previous studies [16–19] have shown that it is difficult to identify angle structures in 3–36.8% of images taken by automated gonioscopy. In our study, only 74 pictures (6.6%) were categorized as blurred images. This result was the same as or slightly better than those reported by previous studies [16, 19]. We instructed our optometrists to acquire images only after the angle structure was confirmed at the time of acquisition, which might have led to more legible images.

We evaluated the correlation between clinical characteristics, such as visual acuity and visual field, in individual cases and the examination time of automated gonioscopy. The results showed a significant positive correlation only for visual acuity in the examined eyes. Furthermore, there were no significant differences in other clinical characteristics between the long- and short-time groups. These results suggest that better visual acuity in the examined eye might have led to better eye fixation and a shorter examination time.

The EyeCam (Clarity Medical Systems, Pleasanton, GA) can obtain gonioscopic images similar to those obtained by automated gonioscopy. EyeCam has been reported to be as useful as gonioscopy in detecting angle closures [20, 21], and the results appear to be comparable for the detection of angle closures with GS-1 [19]. However, although EyeCam is minimally invasive, the examination time of EyeCam was reported to be 5–10 min/eye [20]. Therefore, automated gonioscopy, which completes the examination in about one min, is considered more clinically useful than EyeCam. A comparative study is needed in the future.

Automated gonioscopy allows gonioscopic images to be obtained quickly and minimally invasively. However, GS-1 has several limitations. Automated gonioscopy cannot perform dynamic examinations, including compression. Therefore, there are some cases in which it is not possible to determine whether the angle structure can be judged due to an angle closure or poor image quality using only automated gonioscopy. In addition, some patients with poor eye fixation or difficulty in maintaining posture cannot undergo automated gonioscopy examinations. These findings suggest that automated gonioscopy is not a complete replacement for manual gonioscopy. Manual gonioscopy provides a more detailed view of the angular structure. On the other hand, automated gonioscopy can take 360-degree gonioscopic images, which may be useful for evaluating the changes over time, especially after surgery such as trabeculotomy and trabeculectomy, and for screening for angle closure.

There are some limitations to this study. First, the patients had already undergone gonioscopy several times before participating in the study. If only cases with no history of gonioscopy had been considered, discomfort and pain scores may have been similar between automated and manual gonioscopy owing to the lack of experience of invasive examination with instrumentation touching the ocular surface. A history of gonioscopy examination is a confounding factor, and further study in patients with no history of gonioscopy is warranted. Second, we did not evaluate the learning curve associated with manual gonioscopy. The comparison of the learning curve between automated gonioscopy and manual gonioscopy is also necessary to assess the utility of automated gonioscopy in future studies. Third, manual gonioscopy was performed by a glaucoma specialist and ophthalmology resident while, automated gonioscopy was performed by orthoptists in this study. These were conducted with the assumption of actual users in Japan. The subjective factors such as pain and discomfort are likely to be influenced by the examiner’s technique. To avoid this bias, a detailed examination of a large number of cases would be necessary in the future. Furthermore, there might be bias due to the order of examinations because all cases were examined first by manual gonioscopy and then by automated gonioscopy.

In conclusion, the current study showed that automated gonioscopy is performed with a similar level of invasiveness and examination time as glaucoma specialists. This may improve the quality of glaucoma treatment and research because automated gonioscopy enables us to compare angle structures over time by acquiring 360-degree gonioscopic images.
